# Establishment of Early Multi-Indicator Prediction Models of Moderately Severe Acute Pancreatitis and Severe Acute Pancreatitis

**DOI:** 10.1155/2022/5142473

**Published:** 2022-04-04

**Authors:** Shan-Shan He, Dan Li, Qi-Yong He, Xiao-Ping Chen, Yong-Xu Lin, Yun-Wen Yu, Feng-Lin Chen, Jian Ding

**Affiliations:** ^1^Liver Research Center, The First Affiliated Hospital of Fujian Medical University, Fuzhou 350005, China; ^2^Digestive Department, Union Hospital of Fujian Medical University, Fuzhou 350001, China; ^3^Digestive Department, The First Affiliated Hospital of Fujian Medical University, Fuzhou 350005, China; ^4^Department of Statistics, College of Mathematics and Informatics & FJKLMAA, Fujian Normal University, Fuzhou 350117, China; ^5^Digestive Department, Minnan branch, The First Affiliated Hospital of Fujian Medical University, Quanzhou 362800, China

## Abstract

**Background:**

It is critical to accurately identify patients with severe acute pancreatitis (SAP) and moderately SAP (MSAP) in a timely manner. The study was done to establish two early multi-indicator prediction models of MSAP and SAP.

**Methods:**

Clinical data of 469 patients with acute pancreatitis (AP) between 2015 and 2020, at the First Affiliated Hospital of Fujian Medical University, and between 2012 and 2020, at the Affiliated Union Hospital of Fujian Medical University, were retrospectively analyzed. The unweighted predictive score (unwScore) and weighted predictive score (wScore) for MSAP and SAP were derived using logistic regression analysis and were compared with four existing systems using receiver operating characteristic curves.

**Results:**

Seven prognostic indicators were selected for incorporation into models, including white blood cell count, lactate dehydrogenase, C-reactive protein, triglyceride, D-dimer, serum potassium, and serum calcium. The cut-offs of the unwScore and wScore for predicting severity were set as 3 points and 0.513 points, respectively. The unwScore (AUC = 0.854) and wScore (AUC = 0.837) were superior to the acute physiology and chronic health evaluation II score (AUC = 0.526), the bedside index for severity in AP score (AUC = 0.766), and the Ranson score (AUC = 0.693) in predicting MSAP and SAP, which were equivalent to the modified computed tomography severity index score (AUC = 0.823).

**Conclusions:**

The unwScore and wScore have good predictive value for MSAP and SAP, which could provide a valuable clinical reference for management and treatment.

## 1. Introduction

Acute pancreatitis (AP) refers to intrapancreatic trypsinogen activation caused by gallstones, hypertriglyceridemia, and alcohol abuse, followed by local pancreatic inflammation as the major clinical feature, with or without dysfunction of other organs [1, 2]. AP is the most common pancreatic disease worldwide [3]. Global incidence and mortality are estimated at 33.74 cases (95% confidence interval (CI): 23.33–48.81) per 100 000 person-years and 1.60 deaths (95% CI: 0.85–1.58) per 100,000 person-years [3]. The incidence appears to be rising continuously at an annual rate of 3.4% [4–7]. Although approximately 80–85% of patients with AP experience a mild disease course and have a good prognosis (self-limited, mortality < 3%), 15–20% develop moderately severe AP (MSAP) or severe AP (SAP) [4, 8–11], which have mortality rates as high as 30% [8, 12, 13]. Therefore, it is critical to accurately identify patients with SAP and MSAP in a timely manner.

In 1974, Ranson et al. [14] assessed the severity of AP for the first time. Since then, a series of clinical scoring systems have been gradually developed to stratify the severity and predict the prognosis of AP [15–17], including the Ranson score, the Glasgow score, the acute physiology and chronic health evaluation (APACHE) II score, the bedside index for severity in AP (BISAP), and the modified computed tomography severity index (MCTSI) [9, 17–19]. Nevertheless, both the Ranson score and the Glasgow score require 48 h to be determined. Therefore, the risk stratification cannot be performed at admission, leading to missing a potentially valuable and early therapeutic window. Furthermore, some parameters used in the Ranson score are not routinely collected at the time of hospitalization [9, 20, 21]. The APACHE II score has complex parameters, tedious operation, and poor predictive value within 24 h of onset [9, 21–24]. Wu et al. [21] pointed out that in their validation cohort, only 2.2% of cases had complete APACHE II data. Moreover, whether the APACHE II score reflects the presence of tissue necrosis remains a matter of debate [6]. Unfortunately, pancreatic parenchyma necrosis may not occur in the early stage of the disease, which restricts the early assessment of the severity of AP by MCTSI [10]. In contrast, the BISAP score, which can well predict the severity of AP, organ failure (OF), and death, is easier to calculate and can be evaluated by routine clinical data within 24 h [9, 21]. However, it has also been reported that its sensitivity to mortality and SAP is not ideal [25].

Early prediction of the severity of AP remains a huge challenge. In view of the shortcomings of current risk stratification tools, the purpose of this study is to explore the prognostic factors related to the severity of AP and to establish early multi-indicator prediction models, in order to provide a reference for clinical diagnosis and treatment of AP, early intervention, and hierarchical management.

## 2. Methods

### 2.1. Object of Study

Clinical data of 469 patients who met the inclusion criteria between January 1st, 2015, and June 30th, 2020, at the First Affiliated Hospital of Fujian Medical University, and between January 1st, 2012, and October 31st, 2020, at the Affiliated Union Hospital of Fujian Medical University, were retrospectively collected and analyzed, including 202 cases of mild AP (MAP), 240 cases of MSAP, and 27 cases of SAP.

### 2.2. Diagnostic Criteria and the Revised Atlanta Classification of AP [26]

According to the 2012 revision of the Atlanta classification and definitions by international consensus, the diagnosis of AP requires two of the following three features: (i) abdominal pain consistent with AP (acute onset of a persistent, severe, epigastric pain often radiating to the back); (ii) serum lipase activity (or amylase activity) at least three times greater than the upper limit of normal; and (iii) characteristic findings of AP on contrast-enhanced CT and less commonly magnetic resonance imaging or transabdominal ultrasonography.

1. MAP: MAP is characterized by the absence of local or systemic complications and OF.
2. MSAP: MSAP is characterized by OF that resolves within 48 h (transient OF) and/or local or systemic complications without persistent OF.
3. SAP: SAP is characterized by persistent OF (>48 h), including single OF and multiple OF.

OF is defined as a modified Marshall score ≥ 2 for the renal, respiratory, and/or cardiovascular system. Local complications are AP fluid collection, pancreatic pseudocyst, acute necrotic collection, walled-off necrosis, gastric outlet dysfunction, splenic and portal vein thrombosis, and colonic necrosis. Systemic complications are systemic inflammatory response syndrome (SIRS), OF, sepsis, intra-abdominal hypertension, abdominal compartment syndrome, and pancreatic encephalopathy.

SIRS criteria are as follows: (1) temperature > 38°C or <36°C, (2) respiratory rate > 20 breaths/minute or PaCO_2_ < 32 mmHg, (3) pulse > 90 beats/minute, and (4) white blood cell count (WBC) < 4000 cells/mm^3^ or >12,000 cells/mm^3^ or >10% immature bands. SIRS is defined as the presence of 2 or more SIRS criteria.

### 2.3. Inclusion and Exclusion Criteria

Inclusion criteria were as follows: (1) patients who met the diagnostic criteria of AP, and had complete clinical data, (2) patients who were more than 18 years old, (3) patients in whom systemic examination and abdominal CT scanning were completed, and (4) initial onset.

Exclusion criteria were as follows: (1) patients who were less than 18 years old, (2) acute attack or recurrence of chronic pancreatitis, (3) pregnant or lactating patients, (4) AP caused by malignant tumor or abdominal space occupying lesion, (5) patients who had severe mental/neurological disorders or lack of self-awareness, and (6) whose clinical data were incomplete.

### 2.4. Research Indicators

The statistical data collected in this study include (1) the general clinical data of the patients, including sex, age, etiological type, vital signs, severity score, basic diseases, hospitalization days, and hospitalization expenses and (2) clinical indexes, including WBC, hemoglobin (HB), hematocrit (HCT), platelet (PLT), red blood cell volume distribution width (RDW), RDW/PLT, glucose (GLU), total bilirubin (TBIL), aspartate aminotransferase (AST), lactate dehydrogenase (LDH), albumin (ALB), blood urea nitrogen (BUN), serum creatinine (sCr), the BUN/sCr ratio, total cholesterol (TCHOL), procalcitonin (PCT), bicarbonate ion (HCO_3_^−^), triglyceride (TG), C-reactive protein (CRP), D-dimer, serum calcium, corrected calcium, serum phosphorus, serum sodium, and serum potassium.

### 2.5. Outcome Measurements

Secondary outcome measurements are as follows: the patient data were subjected to univariate analysis and binary logistic regression analysis to obtain independent prognostic indicators of the severity of AP.

Primary outcome measurements are as follows: using the above indicators, unweighted predictive score (unwScore) and weighted predictive score (wScore) for MSAP and SAP were established. The receiver operator characteristic (ROC) curves of independent prognostic factors, 4 existing systems, and prediction models were produced. The area under the curves (AUC) were compared while the cutoff values, the sensitivity, the specificity, the positive predictive values (PPV), the negative predictive values (NPV), and the accuracy rates were calculated to verify the predictive efficiency of new models.

### 2.6. Statistical Analysis

SPSS 24 software and MedCalc v19.3.0 software were used for statistical analysis, and GraphPad Prism 8 was used for drawing. The chi-square test or the Fisher exact test was used for countable data. Normally distributed data are expressed as the mean ± standard deviation ($\bar{x}\pm S$), and the independent sample *t*-test was used for the comparison between groups. Nonnormally distributed data are expressed as the median (quartile spacing), and the Mann–Whitney rank sum test was used for comparison between groups. Binary logistic regression analysis was used to analyze the influencing factors. *P* < 0.05 was considered to indicate statistical significance. ROC curves were used to determine the best critical value of each prognostic index and prediction model and their prediction values.

## 3. Results

### 3.1. Results of Univariate Analysis of Influencing Factors in the MAP and MSAP+SAP Groups

#### 3.1.1. Comparison of General Data between the MAP and MSAP+SAP Groups

There was no significant difference in sex, vital signs (systolic blood pressure, diastolic blood pressure, and mean arterial pressure), APACHE II score, and hypertension between the two groups. There was a significant difference in age, etiology, vital signs (temperature, pulse rate, and respiratory rate), severity score (BISAP score, MCTSI score, and Ranson score), underlying diseases (diabetes, fatty liver, and hyperlipidemia), hospitalization days, and hospitalization expenses (*P* < 0.05) (Table 1).

#### 3.1.2. Comparison of Clinical Indexes between the MAP and MSAP+SAP Groups

There was no significant difference in PLT, RDW/PLT, TBIL, BUN, sCr, and BUN/sCr between the two groups (*P* > 0.05). There was a significant difference in WBC, HB, HCT, RDW, GLU, AST, LDH, ALB, TCHOL, TG, D-dimer, HCO_3_^−^, CRP, PCT, serum calcium, corrected calcium, serum phosphorus, serum sodium, and serum potassium (*P* < 0.05) (Table 2).

### 3.2. Multivariate Analysis of Independent Prognostic Factors in Patients with AP

The clinical indexes WBC, HB, HCT, PLT, RDW, RDW/PLT, GLU, TBIL, AST, LDH, ALB, BUN, sCr, BUN/sCr, TCHOL, TG, D-dimer, HCO_3_^−^, CRP, PCT, serum calcium, corrected calcium, serum phosphorus, serum sodium, and serum potassium were included in the binary logistic regression analysis. The results showed that WBC, LDH, CRP, sCr, TG, D-dimer, and serum potassium were independent prognostic risk factors for MSAP and SAP, while serum calcium was an independent prognostic protective factor for MSAP and SAP (Table 3).

## 4. Establishment of Multi-Index Joint Prediction Models

### 4.1. Determination of the Critical Values of Independent Prognostic Indicators

**Figure 1:**
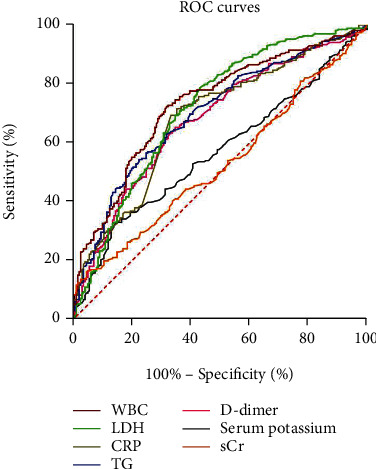
ROC curves of independent prognostic risk factors for AP. Note: AP: acute pancreatitis; ROC: receiver operating characteristic; WBC: white blood cell; LDH: lactate dehydrogenase; CRP: C-reactive protein; TG: triglycerides; sCr: serum creatinine.

**Figure 2:**
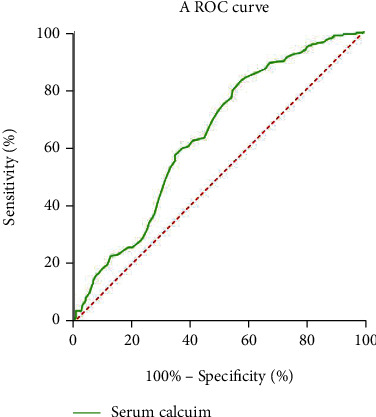
A ROC curve of an independent prognostic protective factor for AP. Note: AP: acute pancreatitis; ROC: receiver operating characteristic.

The data were divided into the MAP group and the MSAP+SAP group, and the critical values of independent prognostic indexes for predicting MSAP+SAP were calculated on the basis of ROC curves. The results showed that WBC, LDH, CRP, TG, D-dimer, serum potassium, and serum calcium had predictive value for MSAP+SAP (Table 4, Figures 1 and 2), which were included in the unwScore model and the wScore model.

### 4.2. unwScore Model

According to the critical value of each index, the continuous variables were transformed into binary variables: WBC > 11.49 × 10^9^/L was defined as 1, WBC ≤11.49 × 10^9^/L as 0; LDH > 246 U/L was defined as 1, LDH ≤ 246 U/L as 0; CRP > 84.05 mg/L was defined as 1, CRP ≤ 84.05 mg/L as 0; TG > 2.01 mM was defined as 1, TG ≤ 2.01 mM as 0; D‐dimer > 2.23 mg/L was defined as 1, D‐dimer ≤ 2.23 mg/L as 0; serum potassium > 4.27 mM was defined as 1, serum potassium ≤ 4.27 mM as 0; and serum calcium ≤ 1.98 mM was defined as 1, serum calcium > 1.98 mM as 0.

On the basis of the critical value of each index, the risk score of MSAP+SAP in patients with AP was calculated according to Equation (1). The critical value of the model for predicting the occurrence of MSAP and SAP in patients with AP was 3. With the increasing model score, the probability of occurrence of MSAP and SAP increased. The probabilities of occurrence of MSAP+SAP corresponding to risk scores of 0, 1, 2, 3, 4, 5, 6, and 7 were 8.82%, 7.14%, 36.11%, 53.47%, 80.95%, 87.14%, 97.50%, and 100%, respectively.

(1) $$\begin{array}{c} \text{unwScore}=S_{\text{WBC}}+S_{\text{LDH}}+S_{\text{CRP}}+S_{\text{TG}}+S_{\text{D}‐\text{dimer}}+S_{\text{serum potassium}}+S_{\text{serum calcium}}. \end{array}$$

**Figure 3:**
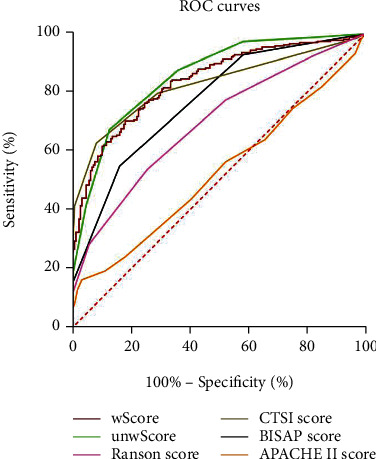
ROC curves of the unweighted predictive model, the weighted predictive model, and the severity scores of AP. Note: AP: acute pancreatitis; ROC: receiver operating characteristic; unwScore: unweighted predictive score; wScore: weighted predictive score; APACHE II: acute physiology and chronic health evaluation II; BISAP: bedside index for severity in acute pancreatitis; MCTSI: modified computed tomography severity index.

The unwScore model was superior to the APACHE II score, the BISAP score, and the Ranson score in predicting the occurrence of MSAP and SAP in patients with AP, which was similar to that of MCTSI (Table 5, Figure 3).

### 4.3. wScore Model

Defining MSAP and SAP as 1 and MAP as 0, binary logistic regression analysis was performed on WBC, LDH, CRP, TG, D-dimer, serum potassium, and serum calcium. The following regression equation (Equation (2)) was established (Table 6). The likelihood ratio test showed that the model was statistically significant (*χ*^2^ = 182.132, *P* ≤ 0.001), while the Hosmer–Lemeshow test indicated that the goodness of fit was good (*χ*^2^ = 5.122, *P* = 0.744).

(2) $$\begin{array}{c} \text{wScore}=-3.420+0.195\times {\text{NV}}_{\text{WBC}}+0.001\times {\text{NV}}_{\text{LDH}}+0.004\times {\text{NV}}_{\text{CRP}}-1.435\times {\text{NV}}_{\text{serum calcium}}+0.070\times {\text{NV}}_{\text{TG}}+0.174\times {\text{NV}}_{\text{D}‐\text{dimer}}+0.667\times {\text{NV}}_{\text{serum potassium}}\\ \text{where NV is the numerical value}. \end{array}$$

The wScore model was superior to the APACHE II score, the BISAP score, and the Ranson score in predicting the occurrence of MSAP+SAP in patients with AP, which was similar to the MCTSI score and the unwScore model (Table 5, Figure 3).

## 5. Discussion

AP is an inflammatory disease of highly variable severity, ranging from mild cases with low mortality to severe cases with high mortality [27]. Early severity stratification becomes critical, especially on the day of admission, as this period is regarded as a window of opportunity to prevent pancreatic necrosis and OF [9]. Currently, there are four frequently used AP scoring systems to assist clinicians to identify SAP and MSAP, namely, the APACHE II score, the Ranson score, the BISAP score, and the MCTSI score. Each scoring system has specific applications and advantages, but each also has limitations.

In the present study, we constructed early multi-indicator prediction models that comprise only seven prognostic indexes: WBC, LDH, CRP, TG, D-dimer, serum potassium, and serum calcium. Compared with the traditional scoring systems, the advantages of the unwScore and wScore models constructed in this study lie in their simplicity, safety, objectivity, low cost, early risk stratification, high predictive value, dynamic monitoring, and quantitative prediction.

In 1974, Ranson et al. [14] first put forward the Ranson score, which is a milestone in the assessment of AP severity. However, the Ranson score is based on 11 parameters identified as important prognostic factors, requiring 48 hours for a complete evaluation [28]. Similarly, it is only applicable within 48 hours of admission, and it is difficult to track and evaluate the changes of the disease in real time [29]. Different from the Ranson score, the unwScore and wScore models could be used simultaneously by using only seven variables within 24 hours after admission. Similarly, the unwScore model could also quantitatively predict the occurrence probability of MSAP and SAP. With the increasing model score, the probability of occurrence of MSAP and SAP increased.

In 2004, Mortele et al. [30] revised and simplified the CTSI score and put forward the MCTSI score, which correlated more closely with the severity of AP and the following parameters: the length of the hospital stay, the need for surgical or percutaneous procedures, and the occurrence of infection. However, the ideal time for CT scans is at least 72 hours after the onset of symptoms [31], and it is not recommended to conduct routine CT scans on admission purely for the purpose of severity assessment [32]. Enhanced CT scans are also not recommended when there is significant renal damage (usually creatinine levels higher than 1.5 mg/dL) or a history of obvious allergy to contrast media [33]. Compared with the MCTSI score, the advantages of our models are that they are not only simple and fast but also does not need imaging examination, thus reducing unnecessary radiation and economic burden. Especially when severe and unstable patients are not suitable for CT enhanced scanning, it is not conducive to the early evaluation of MCTSI score.

The APACHE score was originally designed to assess the severity of acute illness patients who entered the ICU in the 1970s [9]. In 1985, Knaus et al. [22] simplified the APACHE score and proposed the APACHE II score, which is the most commonly used. Still, the APACHE II score takes into account various parameters, including acute physiological variables, age, and chronic health conditions, some of which may not be relevant to AP prognosis, whereas other important measures, such as pancreatic injury and significant regional complications, are missed [21, 33, 34]. Unlike the APACHE II score, the seven prognostic indicators included in the unwScore and wScore models all play a key role in the occurrence and development of AP or have clinical significance [9, 20, 27, 35–52]. These indexes are easy to obtain in the clinic, and the detection method is convenient. Furthermore, the model algorithm is simple, rapid, feasible, and easy to popularize and monitor dynamically and is also suitable for grass-roots hospitals. Beyond that, the unwScore and wScore models can also avoid unnecessary pain and financial expenditure to patients because blood gas analysis is not necessary.

In 2008, Wu et al. [21] proposed the BISAP score. The detail of “SIRS” in the system is mainly based on the values of vital signs, which makes the score unstable so that evaluation has to be conducted repeatedly. And similar to APACHE II score, the criterion of “impaired mental status” in this system is subjective. Fortunately, all the variables included in the unwScore and wScore models are objective routine clinical indicators, thus reducing the subjective bias of evaluators, which could be used between different centers.

Compared to the four existing scoring systems, the performance of new models was good for the prediction of AP severity. The overall sensitivity, specificity, PPV, and NPV for the prediction of disease severity with the Ranson score were 75%, 77%, 49%, and 91%, respectively, according to a meta-analysis. At admission, the sensitivity of the APACHE II score > 7 to predict SAP is 65%, with a specificity of 76%, a PPV of 43%, a NPV of 89%, and an accuracy of 75%. At 48 hours, the sensitivity of the APACHE II score > 7 to predict SAP is 76%, with a specificity of 84%, a PPV of 54%, a NPV of 93%, and an accuracy of 70–80% [28, 53]. Though the NPVs of our scoring systems were not satisfactory (66.92% and 67.48%), they had similar sensitivity (67.42% and 70.41%) and accuracy (75.91% and 75.05%) and slightly higher specificity (87.13% and 81.19%) and PPV (87.38% and 83.19%). Moreover, compared with the traditional score, although the BISAP score is a big step forward in simplicity, it is not an advancement in accuracy [29]. A prospective study by Papachristou et al. [34] showed that the BISAP score (AUC = 0.81, 95% CI: 0.74–0.87) was similar to the Ranson score (AUC = 0.94, 95% CI: 0.89–0.97), the APACHE II score (AUC = 0.78, 95% CI: 0.71–0.84), and the MCTSI score (AUC = 0.84, 95% CI: 0.76–0.89) in predicting SAP. While preserving their advantages of simplicity and efficiency, the unwScore (AUC = 0.854) and wScore (AUC = 0.837) models constructed in this study could still predict the severity of AP well, and their predictive values were even superior to those of the BISAP score (AUC = 0.766), the APACHE II score (AUC = 0.526), and the Ranson score (AUC = 0.693) and equivalent to that of the MCTSI score (AUC = 0.823).

In summary, we have derived two new scoring systems for predicting severity of AP based on commonly used clinical indexes. All seven variables included in this scoring system can be easily measured within 24 hours of admission. Compared to four existing scoring systems, the new prediction models are accurate in predicting disease severity (MSAP and SAP), which plays important roles in early evaluation, progress analysis, treatment plan adjustment, and prognosis judgment of AP.

## 6. Conclusions

WBC, LDH, CRP, sCr, TG, D-dimer, and serum potassium are independent prognostic risk factors for the severity of AP, while serum calcium is an independent prognostic protective factor. The early multi-indicator prediction models of MSAP and SAP have a good predictive efficiency and could provide a valuable clinical reference for prediction and treatment.

## Figures and Tables

**Table 1:** Comparison of general data between the MAP and MSAP+SAP groups.

		MAP (202 cases)	MSAP+SAP (267 cases)	*χ* ^2^/*Z*/*T*	*P*
Sex	Male, *n* (%)	115 (56.93%)	173 (64.79%)	3.000	0.083
	Female, *n* (%)	87 (43.07%)	94 (35.21%)		
Age (years)		58 (46, 70)	45 (36,62)	−5.999	≤0.001
Etiology	Biliary, *n* (%)	140 (69.31%)	113 (42.32%)	39.764	≤0.001
	Hyperlipidemic, *n* (%)	32 (15.84%)	109 (40.82%)		
	Alcoholic, *n* (%)	13 (6.44%)	21 (7.87%)		
	Other, *n* (%)	17 (8.42%)	24 (8.99%)		
Vital signs	Temperature (°C)	36.6 (36.5, 36.8)	36.8 (36.5, 37.3)	−5.608	≤0.001
	Pulse rate (times/minute)	77 (69, 82)	97 (82, 108)	−12.039	≤0.001
	Respiratory rate (times/minute)	19 (18, 20)	20 (19, 21)	−7.469	≤0.001
	Systolic blood pressure (mmHg)	130 (116, 145)	131 (120, 145)	−1.114	0.265
	Diastolic blood pressure (mmHg)	80 (70, 87)	80 (71, 90)	−1.043	0.297
	Mean arterial pressure (mmHg)	96.678 ± 13.985	98.157 ± 14.577	1.107	0.269
Score	APACHE II	6 (3.75, 8)	6 (3, 8)	−0.955	0.339
	BISAP	1 (0, 1)	2 (1, 2)	−10.415	≤0.001
	MCTSI	2 (2, 4)	6 (4, 8)	−12.468	≤0.001
	Ranson	2 (1, 3)	3 (2, 4)	−7.309	≤0.001
Underlying diseases	Hypertension, *n* (%)	68 (33.66%)	81 (30.34%)	0.587	0.444
	Diabetes, *n* (%)	36 (17.82%)	84 (31.46%)	11.397	0.001
	Fatty liver, *n* (%)	67 (33.17%)	131 (49.06%)	11.911	0.001
	Hyperlipidemia, *n* (%)	50 (24.75%)	136 (50.94%)	32.946	≤0.001
Hospitalization days (days)		10 (8, 13)	12 (9, 16)	−3.592	≤0.001
Hospitalization expenses (yuan)		14841.670 (11016.618, 21544.135)	29289.900 (18598.060, 50166.300)	−10.130	≤0.001

Note: MAP: mild acute pancreatitis; MSAP: moderately severe acute pancreatitis; SAP: severe acute pancreatitis; APACHE II: acute physiology and chronic health evaluation II; BISAP: bedside index for severity in acute pancreatitis; MCTSI: modified computed tomography severity index.

**Table 2:** Comparison of clinical indexes between the MAP and MSAP+SAP groups.

Clinical index	MAP (202 cases)	MSAP+SAP (267 cases)	*Z*	*P*
WBC (10^9^/L)	10.000 (8.000, 12.530)	13.820 (11.250, 17.450)	−8.605	≤0.001
HB (g/L)	133.500 (123.000, 145.000)	142.000 (123.000, 156.000)	−3.322	0.001
HCT (L/L)	0.396 (0.365, 0.426)	0.412 (0.362, 0.447)	−2.200	0.028
PLT (10^9^/L)	210.500 (158.750, 258.750)	212.000 (169.000, 270.000)	−0.894	0.371
RDW (%)	13.000 (12.400, 13.800)	13.200 (12.700, 13.900)	−2.402	0.016
RDW/PLT	0.062 (0.051, 0.084)	0.063 (0.049, 0.079)	−0.778	0.437
GLU (mM)	7.865 (6.213, 9.505)	9.09 (6.720, 12.500)	−4.141	≤0.001
TBIL (*μ*M)	20.500 (13.800, 37.700)	18.600 (11.400, 31.100)	−1.857	0.063
AST (U/L)	46.500 (23.000, 171.250)	37.000 (22.000, 92.000)	−2.249	0.025
LDH (U/L)	225.500 (176.750, 412.250)	423.000 (264.000, 706.000)	−8.001	≤0.001
ALB (g/L)	35.500 (32.850, 38.525)	33.800 (30.800, 38.100)	−3.271	0.001
BUN (mM)	4.400 (3.300, 5.700)	4.500 (3.330, 6.370)	−1.502	0.133
sCr (*μ*M)	65.000 (50.425, 78.000)	65.000 (51.000, 81.400)	−1.040	0.298
BUN/sCr	0.067 (0.053, 0.088)	0.069 (0.052, 0.088)	−0.129	0.898
TCHOL (mM)	4.160 (3.318, 5.195)	4.640 (3.440, 7.210)	−3.502	≤0.001
TG (mM)	1.065 (0.768, 1.858)	2.320 (1.080, 8.930)	−7.423	≤0.001
D-dimer (mg/L)	1.670 (0.890, 2.973)	2.990 (1.600, 5.720)	−6.633	≤0.001
HCO_3_^−^ (mM)	23.750 (21.300, 26.000)	21.900 (18.800, 24.200)	−5.706	≤0.001
CRP (mg/L)	61.195 (21.808, 90.000)	90.000 (72.700, 172.000)	−6.875	≤0.001
PCT (ng/mL)	0.176 (0.064, 0.839)	0.640 (0.210, 2.280)	−6.017	≤0.001
Serum calcium (mM)	2.120 (2.010, 2.223)	2.020 (1.900, 2.180)	−5.196	≤0.001
Corrected calcium (mM)	2.197 (2.111, 2.291)	2.132 (2.018, 2.248)	−4.460	≤0.001
Serum phosphorus (mM)	138.600 (136.275, 140.800)	137.100 (134.600, 139.400)	−4.346	≤0.001
Serum sodium (mM)	3.880 (3.610, 4.150)	4.010 (3.620, 4.410)	−2.530	0.011
Serum potassium (mM)	0.810 (0.618, 0.973)	0.740 (0.520, 0.970)	−2.022	0.043

Note: MAP: mild acute pancreatitis; MSAP: moderately severe acute pancreatitis; SAP: severe acute pancreatitis; WBC: white blood cell; HB: hemoglobin; HCT: hematocrit; PLT: platelet; RDW: red blood cell volume distribution width; GLU: glucose; TBIL: total bilirubin; AST: aspartate aminotransferase; LDH: lactate dehydrogenase; ALB: albumin; BUN: blood urea nitrogen; sCr: serum creatinine; TCHOL: total cholesterol; TG: triglycerides; HCO_3_^−^: bicarbonate ion; CRP: C-reactive protein; PCT: procalcitonin.

**Table 3:** Logistic regression analysis of independent prognostic factors in MSAP and SAP.

	*B*	Standard error	Wald	*P*	OR	95% CI	
						Lower limit	Upper limit
WBC	0.201	0.030	44.819	≤0.001	1.223	1.153	1.297
LDH	0.001	0.000	8.503	0.004	1.001	1.000	1.001
CRP	0.004	0.001	7.813	0.005	1.004	1.001	1.007
Serum calcium	−1.450	0.615	5.567	0.018	0.235	0.070	0.782
sCr	0.006	0.003	4.188	0.041	1.006	1.000	1.012
TG	0.075	0.023	10.702	0.001	1.078	1.031	1.127
D-dimer	0.170	0.046	13.902	≤0.001	1.186	1.084	1.297
Serum potassium	0.598	0.230	6.793	0.009	1.819	1.160	2.853
Constant	−3.636	1.604	5.138	0.023	0.026		

Note: MSAP: moderately severe acute pancreatitis; SAP: severe acute pancreatitis; WBC: white blood cell; LDH: lactate dehydrogenase; CRP: C-reactive protein; sCr: serum creatinine; TG: triglycerides.

**Table 4:** The predictive values of independent prognostic indexes and predictive models of MSAP and SAP.

	AUC	*P*	Critical value	Sensitivity (%)	Specificity (%)	Positive predictive value	Negative predictive value	Youden index	Accuracy rate (%)
WBC	0.732	<0.0001	>11.49	72.28	68.32	75.10	34.91	0.4060	70.58
LDH	0.716	<0.0001	>246	78.65	56.93	70.71	66.86	0.3558	69.30
CRP	0.684	<0.0001	>84.05	71.54	64.85	72.90	63.29	0.3639	68.66
sCr	0.528	0.299	>106	16.10	95.54	82.69	46.28	0.1165	50.32
TG	0.700	<0.0001	>2.01	54.31	77.72	76.32	56.27	0.3203	64.39
D-dimer	0.679	<0.0001	>2.23	65.17	65.35	71.31	58.67	0.3052	65.25
Serum potassium	0.568	0.0097	>4.27	32.58	85.64	75.00	49.01	0.1823	55.44
Serum calcium	0.640	<0.0001	≤1.98	42.32	83.17	76.87	52.17	0.2549	59.91
unwScore	0.854	<0.0001	>3	67.42	87.13	87.38	66.92	0.5454	75.91
wScore	0.837	<0.0001	>0.513	70.41	81.19	83.19	67.48	0.5210	75.05

Note: MSAP: moderately severe acute pancreatitis; SAP: severe acute pancreatitis; AUC: area under the ROC curve; WBC: white blood cell; LDH: lactate dehydrogenase; CRP: C-reactive protein; sCr: serum creatinine; TG: triglycerides; unwScore: unweighted predictive score; wScore: weighted predictive score.

**Table 5:** Comparison of the area under the ROC curve of the unweighted prediction model, the weighted prediction model, and the severity score in predicting MSAP and SAP.

	AUC	*P*				
		wScore	APACHE II score	BISAP score	MCTSI score	Ranson score
unwScore	0.854	0.2165	<0.0001	0.0005	0.1635	<0.0001
wScore	0.837		<0.0001	0.0060	0.5680	<0.0001
APACHE II score	0.526			<0.0001	<0.0001	<0.0001
BISAP score	0.766				0.0110	0.0028
MCTSI score	0.823					<0.0001
Ranson score	0.693					

Note: MSAP: moderately severe acute pancreatitis; SAP: severe acute pancreatitis; AUC: area under the ROC curve; unwScore: unweighted predictive score; wScore: weighted predictive score; APACHE II: acute physiology and chronic health evaluation II; BISAP: bedside index for severity in acute pancreatitis; MCTSI: modified computed tomography severity index.

**Table 6:** Results of logistic regression analysis of independent prognostic factors for MSAP and SAP.

	*B*	Standard error	Wald	*P*	OR	95% CI	
						Lower limit	Upper limit
WBC	0.195	0.029	44.087	≤0.001	1.215	1.147	1.287
LDH	0.001	0.000	9.075	0.003	1.001	1.000	1.001
CRP	0.004	0.001	8.082	0.004	1.004	1.001	1.007
TG	0.070	0.022	9.593	0.002	1.072	1.026	1.120
D-dimer	0.174	0.046	14.467	≤0.001	1.190	1.088	1.301
Serum potassium	0.667	0.225	8.777	0.003	1.948	1.253	3.029
Serum calcium	−1.435	0.601	5.708	0.017	0.238	0.073	0.773
Constant	−3.420	1.569	4.753	0.029	0.033		

Note: MSAP: moderately severe acute pancreatitis; SAP: severe acute pancreatitis; WBC: white blood cell; LDH: lactate dehydrogenase; CRP: C-reactive protein; TG: triglycerid.

## Data Availability

The data used to support the findings of this study are available from the corresponding author upon request.
